# Head-Movement-Emphasized Rehabilitation in Bilateral Vestibulopathy

**DOI:** 10.3389/fneur.2018.00562

**Published:** 2018-07-17

**Authors:** Nadine Lehnen, Silvy Kellerer, Alexander G. Knorr, Cornelia Schlick, Klaus Jahn, Erich Schneider, Maria Heuberger, Cecilia Ramaioli

**Affiliations:** ^1^Department of Psychosomatic Medicine and Psychotherapy, Klinikum Rechts der Isar,Technical University of Munich, Munich, Germany; ^2^German Center for Vertigo and Balance Disorders, Ludwig Maximilians University, Munich, Germany; ^3^Institute of Medical Technology, Brandenburgische Technische Universität, Cottbus, Germany; ^4^Center for Sensorimotor Research, Ludwig Maximilians University, Munich, Germany; ^5^Department of Electrical and Computer Engineering, Institute for Cognitive Systems, Technical University of Munich, Munich, Germany; ^6^Department of Neurology,Schoen Clinic Bad Aibling, Bad Aibling, Germany; ^7^Department of Neurology, Ludwig Maximilians University, Munich, Germany

**Keywords:** vestibular rehabilitation, bilateral vestibular hypofunction, re-fixation saccades, vestibulo-ocular reflex, HITD-FT, dynamic vision

## Abstract

**Objective:** Although there is evidence that vestibular rehabilitation is useful for treating chronic bilateral vestibular hypofunction (BVH), the mechanisms for improvement, and the reasons why only some patients improve are still unclear. Clinical rehabilitation results and evidence fromeye-head control in vestibular deficiency suggest that headmovement is a crucial element of vestibular rehabilitation. In this study, we assess the effects of a specifically designed head-movement-based rehabilitation program on dynamic vision, and explore underlying mechanisms.

**Methods:** Two adult patients (patients 1 and 2) with chronic BVH underwent two 4-week interventions: (1) head-movement-emphasized rehabilitation (HME) with exercises based on active head movements, and (2) eye-movement-only rehabilitation (EMO), a control intervention with sham exercises without head movement. In a double-blind crossover design, the patients were randomized to first undergo EMO (patient 1) and–after a 4-week washout–HME, and vice-versa (patient 2). Before each intervention and after a 4-week follow-up patients’ dynamic vision, vestibulo-ocular reflex (VOR) gain, as well as re-fixation saccade behavior during passive headmotion were assessed with the head impulse testing device–functional test (HITD-FT).

**Results:** HME, not EMO, markedly improved perception with dynamic vision during passive head motion (HITD-FT score) increasing from 0 to 60% (patient 1) and 75% (patient 2). There was a combination of enhanced VOR, as well as improved saccadic compensation.

**Conclusion:** Head movement seems to be an important element of rehabilitation for BVH. It improves dynamic vision with a combined VOR and compensatory saccade enhancement.

## Introduction

Bilateral vestibular hypofunction (BVH) significantly affects quality of life (1). Patients suffer from symptoms like oscillopsia with head movement and postural instability, leading to difficulties with activities of daily living like driving and a 31-fold increased risk of falls with considerate morbidity (1). Mostly due to ototoxic aminoglycosides, Menière's disease or meningitis (2), BVH has an unfavorable prognosis with no improvement of peripheral vestibular function over several years in more than 80% of patients (3).

At variance with its clinical importance, and compared to most other vestibular disorders (including unilateral vestibular dysfunction), prospective therapeutic clinical trials in patients with BVH are sparse (4, 5). Treatment mostly relies on physical therapy. While it is consensus that vestibular rehabilitation is beneficial [(6–9), for review see (5), for clinical practice guideline see (4)], the mechanisms for improvement, their relative importance and the reasons why only some patients improve are still unclear (6–10).

Clinical rehabilitation results (4–9) and evidence from eye-head control in vestibular deficiency (11–14) suggest that head movement is a crucial element of vestibular rehabilitation. Head motion may improve vestibulo-ocular reflex (VOR) function in BVH (8, 9). Residual vestibular input during head movements is essential for triggering compensatory short-latency re-fixation saccades during passive head movements (12), which, in turn may improve dynamic visual function (13, 14).

In this case study, we assessed whether a specifically designed rehabilitation program based on head motion improves dynamic vision in BVH, and explore underlying mechanisms.

## Materials and methods

### Patients

Two patients (patient 1 and 2, 45–60 years old, gender and exact age have been removed on request of the journal) with chronic BVH were included. They were the only patients who completed the entire proposed program of the study “Eye-Head Movement in Bilateral Vestibulopathy: Translating Optimal Control Modeling and Neurophysiology to Rehabilitation,” a completed translational pilot randomized controlled trial (for details on the study program, see study design below and supplement). In both cases, BVH was due to therapy with ototoxic aminoglycosides while being treated for endocarditis. Clinical BVH symptoms (visual blurring with head movement, and difficulties walking in darkness or on unsteady surfaces) had been present for 9 and 4 months for patients 1 and 2, respectively, slow phase eye movement response on bi-thermal water caloric testing was smaller than 5°/s bilaterally, and there was bilateral vestibular dysfunction in video head impulse testing. There was no clinical manifestation of cerebellar syndrome, polyneuropathy, anxiety or mood disorder in history or clinical neurological and psychiatric examination. Uncompensated vision of the better eye was better than 20% (4/20). None of the patients previously participated in vestibular rehabilitation. The patients did not experience any other changes in activity such as new exercises during the intervention or follow-up.

### Ethics statement

The ethics committee of the Medical Faculty of Ludwig Maximilians University of Munich approved the study, which was conducted in accordance with the principles expressed in the Declaration of Helsinki. All patients gave their written informed consent prior to participation, and were free to withdraw from the study at any time.

### Study design

**Figure 1:**
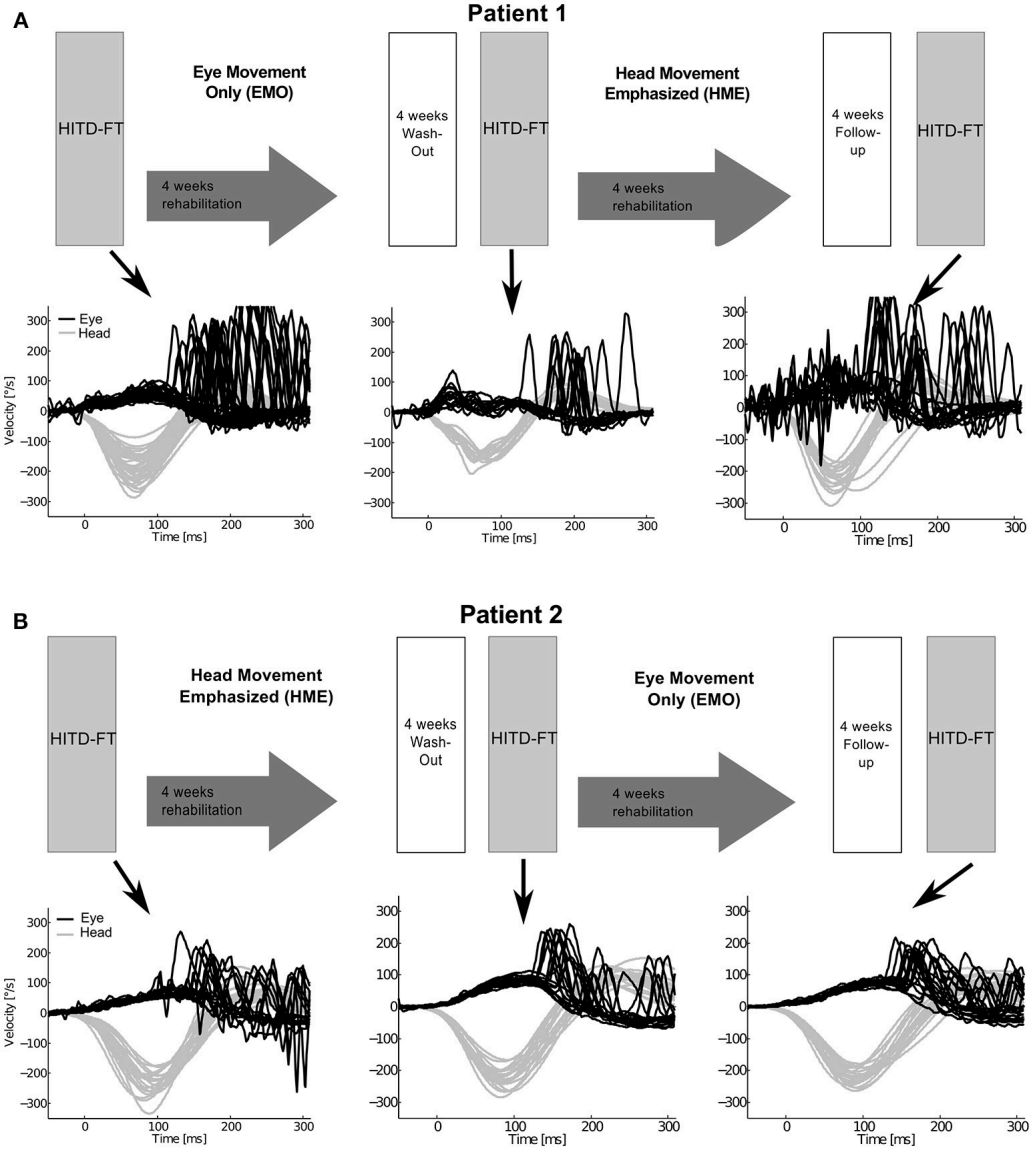
Study design and eye and head movement recordings during head impulse testing device—functional testing (HITD-FT). The upper parts of this figure show the crossover study design for both patients. Patient 1 **(A)** was first treated with eye-movement-only rehabilitation (EMO), and, after a 4-week washout, with head-movement-emphasized rehabilitation (HME), patient 2 **(B)** first with HME, then with EMO. The lower parts of this figure display the corresponding recorded eye and head velocity data during HITD-FT testing with pooled head motion directions. During this test, patients were asked to determine the orientation of a Landolt ring on a screen 2 m straight ahead while their head was passively moved. The head movement is shown in gray, the eye movement in black. Note that vestibulo-ocular reflex (head impulse) gain, i.e., the ratio of median eye and head velocity within a 10-ms-window between 55 and 65 ms after head impulse onset, and compensatory saccade amplitude (integration of the area under the saccade(s) deviating from VOR slow phase velocity) improve after HME, not EMO.

There was a therapeutic randomized controlled double-blind (examiner) crossover design (Figure 1), consisting of two 4-week interventions [Supplement 1, in analogy to (7)]: (1) head-movement-emphasized rehabilitation (HME) with exercises based on active head movements, both during active combined eye-head gaze shifts to a target and during fixation, thereby including a gaze stability task, and (2) eye-movement-only rehabilitation (EMO), a control intervention with eye movement exercises without head movement. Additional information regarding the specifics of each exercise protocol is detailed in the supplement. The crossover design was chosen to avoid possible influencing factors, in particular spontaneous recovery. Patients were randomized to first undergo EMO (patient 1) and—after a 4-week washout—HME, and vice-versa (patient 2). Before each intervention and after a 4-week follow-up dynamic vision, head impulse gain, as well as re-fixation saccade behavior during passive head motion were assessed with the head impulse testing device—functional test (HITD-FT).

### Head impulse testing device—functional test (HITD-FT)

An experienced examiner standing behind the patients performed passive, high-acceleration (3,500–5,000°/s^2^), small amplitude (13–25°) head rotations to the left and right in the plane of the horizontal semicircular canals while patients fixated a standard Landolt ring on a screen 2 m straight ahead [HITD-FT testing in analogy to (13, 15, 16)]. Impulses were delivered with random timing and direction, to prevent anticipation. The size of the Landolt ring during the HITD-FT test was 0.6 logMAR bigger than the static visual acuity test [in analogy to (13)] and remained unchanged during the HITD-FT test. The Landolt ring had a gap measuring of the ring diameter with eight possible gap positions at 45° increments. It appeared on the screen 58 ± 2 ms (mean ± SD) after head velocity reached 20°/s. Display duration was 173 ± 6 ms. Patients had to identify the position of the gap. They provided answers using an external computer keypad consisting of buttons for each gap position. Patients pressed a special “x” button if they had low confidence in their answer to further reduce the possibility of random correct answers. The answer was rated as correct or incorrect (including button x) for each trial. During the HITD-FT, eye movements were recorded by video-oculography of the left eye, head movements by inertial sensors (EyeSeeCam system with a sampling rate of 220 Hz, in analogy to (13, 17).

### Data analysis

Data were analyzed offline using MATLAB (MathWorks, Natick, MA) software. Head impulses and saccades were automatically detected using velocity and acceleration criteria with the possibility for manual correction. Head impulse started when head velocity exceeded 20°/s. Head impulse gain was calculated as the ratio between median eye and head velocity within a 10-ms-window between 55 and 65 ms after head impulse onset. There was no side difference in gain (Wilcoxon sign test, *p* > 0.05), so data from both sides was pooled. On average 22 ± 9 (mean ± SD) trials were considered for analysis. Eye movements within 300 ms after head impulse start characterized by an acceleration higher than 2,000°/s^2^ were considered as re-fixation saccades. An acceleration threshold of 2,000°/s^2^ was used to determine saccade onset, while an acceleration threshold of −2,000°/s^2^ was used to determine saccade offset. Compensatory saccade amplitude deviation from the VOR slow phase velocity was computed by integrating the area under the saccade(s). HITD-FT score was calculated as the rate (percentage) of correct answers from all trials of one patient in one session.

### Statistical analysis

For statistics, not the average, but all the values were used. Normality was assessed by Shapiro-Wilk testing. Differences in head impulse gains between the different orders of treatment were assessed with an independent samples Mann-Whitney-U test, differences within time points (pre- and post-EMO, pre- and post-HME) of both patients were assessed with a related samples Friedman ANOVA-by-ranks. Pairwise comparisons before and after each intervention were assessed by related samples Wilcoxon signed rank testing. All statistical testing was performed on two-sided exploratory 5% significance levels. Computations were conducted with SPSS (SPSS Statistics for Mac).

## Results

**Figure 2:**
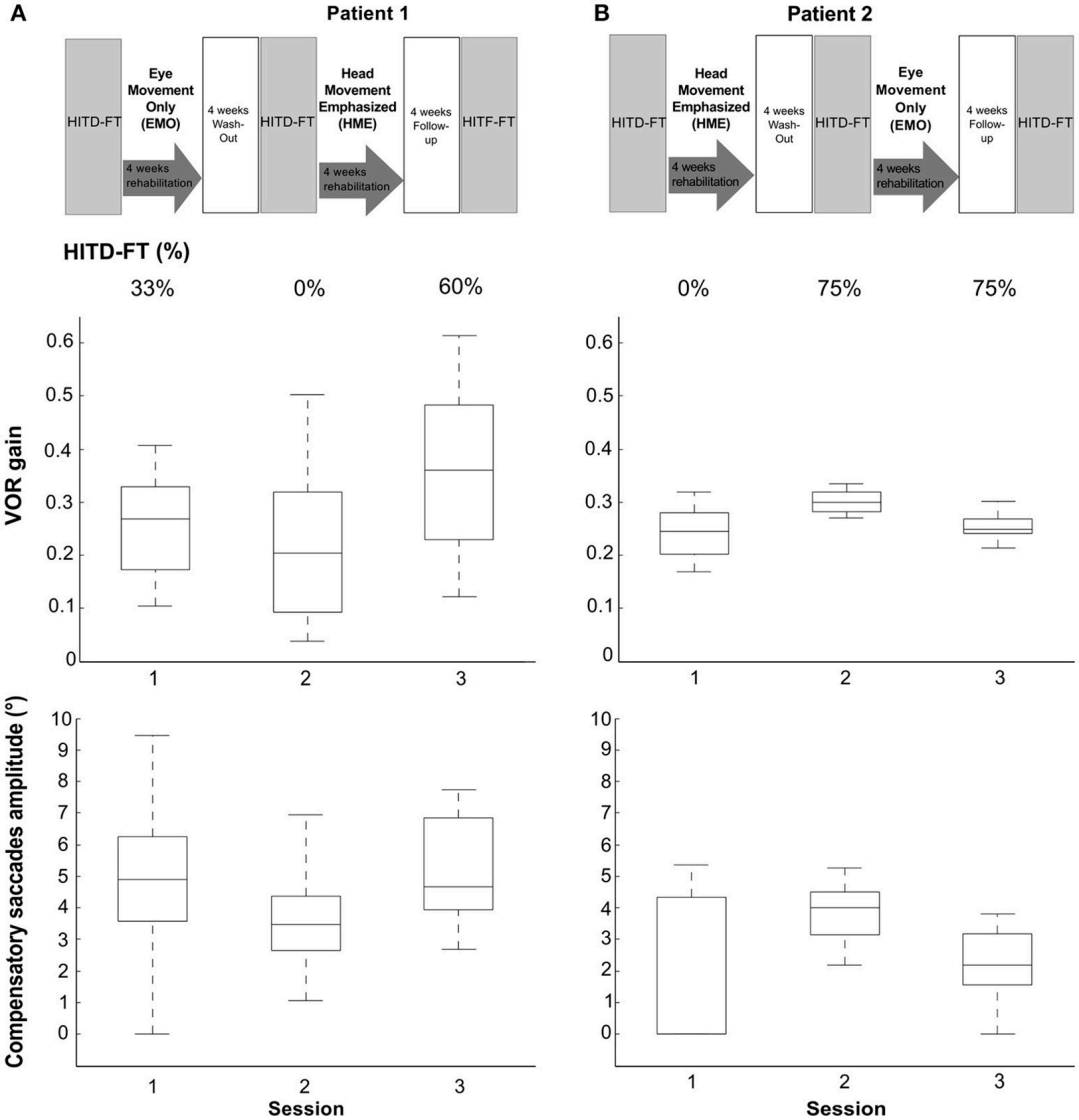
Rehabilitation effects on head impulse testing device—functional testing (HITD-FT) scores, head impulse gain, and compensatory saccade amplitude. This figure shows HITD-FT scores (top), head impulse gain (middle), and compensatory saccade amplitude (bottom) in the course of the rehabilitation program sketched on top for patients 1 **(A)** and 2 **(B)**. In a crossover design, patient 1 was first treated with eye-movement-only rehabilitation (EMO), and, after a 4-week washout, with head-movement-emphasized rehabilitation (HME), patient 2 first with HME, then with EMO. During HITD-FT testing, patients were asked to determine the orientation of a Landolt ring on a screen two meters straight ahead while their head was passively moved. HITD-FT score was calculated as the rate (percentage) of correct answers from all trials of one patient in one session. From simultaneous recordings of eye and head movement, vestibulo-ocular reflex (VOR) gain (ratio of median eye and head velocity within a 10 ms window between 55 and 65 ms after head impulse onset) and saccade amplitude (integration of the area under the saccade(s) deviating from VOR slow phase velocity) were calculated. Gain and saccade amplitude are visualized in box plots. On each box, the central mark is the median, the edges of the box are the 25 and 75th percentiles, the whiskers extend to the most extreme datapoints the algorithm considers to be not outliers. Note the marked increase in dynamic vision (HITD-FT score) after HME with a combined enhancement of the VOR gain and compensatory saccade amplitude in this crossover design in both patients. After EMO, dynamic vision decreased (patient 1) or stayed stable (patient 2) while VOR gain and compensatory saccade amplitude deteriorated in both patients.

Vestibular rehabilitation based solely on head movement exercises (HME) improved dynamic vision, with HITD-FT scores increasing from 0% before HME to 60% (patient 1) and 75% (patient 2) afterwards (Figure 2). With the EMO protocol, dynamic vision decreased (from 33 to 0% in patient 1) or remained stable (75%, patient 2). Figure 1 shows the underlying recorded eye movement behavior with passive head motion. EMO and HME had an effect on both head impulse gains [related samples Friedman ANOVA-by-ranks, patient 1: $\chi_{\left(2\right)}^{2}$ = 43.3, *p* = 0; patient 2: $\chi_{\left(2\right)}^{2}$ = 28.7, *p* = 0] and compensatory saccade amplitude [patient 1: $\chi_{\left(2\right)}^{2}$ = 21.8, *p* = 0; patient 2: $\chi_{\left(2\right)}^{2}$ = 11, *p* = 0.004]. Effects were dependent on the order of the treatments (independent samples Mann-Whitney-U test, *p* < 0.05), therefore, each patient of the crossover design was considered individually. Head impulse gain increased with HME by 80% from 0.2 to 0.36 in patient 1 (Figure 2, Wilcoxon signed-rank test *Z* = −5.05, *p* = 0) and by 20% from 0.25 to 0.3 in patient 2 (*Z* = −4.3, *p* = 0), and it decreased with EMO (patient 1: *Z* = −2.53, *p* = 0.012; patient 2: *Z* = −4.4, *p* = 0). Compensatory saccade amplitude increased with HME (patient 1: *Z* = −5.38, *p* = 0; patient 2: *Z* = −3.18, *p* = 0), and decreased with EMO (patient 1: *Z* = −2.74, *p* = 0.006; patient 2: *Z* = −2.97, p = 0.003).

## Discussion

Head movement seems to be an important element of rehabilitation for BVH. It improves dynamic vision, enhancing both VOR function and compensatory saccade strategies.

Clinically, HITD-FT results of >80% are considered physiological. In this light, an increase from 0%, i.e., the inability to see clearly during head motion, to detecting 60 and 75% of Landolt ring orientation, respectively, after HME, appears practically relevant. Similarly, VOR gain improvements with HME (80% in patient 1 and 20% in patient 2) seem meaningful. They exceed minimal detectable change (MDC) suggested by studies in healthy subjects with MDC values ranging from 11% [repeatability coefficient of 0.1 and average VOR gain of 0.94, (18)] to 14% [95% limits of agreement of 0.14 and average VOR gain of 1.06, (19)].

To our knowledge, this is the first time vestibular rehabilitation based solely on head motion is assessed. The fact that head motion exercises improve dynamic vision in our patients underlines the importance of this part of vestibular rehabilitation. This is very much in line with the clinical practice guidelines, the suggestions from former studies using combined head, balance, and gait exercises (8, 9), and with a study comparing eye and head movement exercises within a comprehensive rehabilitation program (7). Interestingly, both head impulse gain and saccade compensation strategies deteriorated after EMO. This supports the notion expressed in the clinical practice guidelines to avoid isolated saccade or smooth pursuit eye-movement exercises during vestibular rehabilitation (4).

Our case study shows an effect of HME on dynamic vision during high-acceleration, passive unpredictable head motion. This result, which is in line with that of another case study reporting an effect of balance and gaze stabilization exercises on passive dynamic visual acuity (8), is promising, as patients are significantly disabled during activities comprising passive high-frequency head motion like walking or driving (1). In these situations, compensatory strategies such as feed-forward eye movement control [for predictable/active head movements (20, 21)], or smooth-pursuit function [for low head velocities (22)] are not readily available, so that patients have to rely mostly on modification of saccade behavior (14, 23–27), or enhanced vestibular function (8).

As underlying mechanism for the dynamic vision improvement we found both an increase in head impulse gain as well as an increase in compensatory saccade amplitude. This combined effect in a program based on head motion is encouraging, especially because some combined vestibular rehabilitation programs found no significant improvement of VOR function (7), an increase solely of VOR function, but not in compensatory saccades [one BVH patient, balance and gaze stabilization exercises (8)] as well as a combined increase in VOR function and compensatory saccades [one BVH patient, balance, gait and gaze stabilization exercises (9)]. The combined effect could be due to central processing via the cerebellum (10).

This study is clearly limited by the small number of participants. This in mind, it suggests that head movement is an important element of rehabilitation for BVH. It improves dynamic vision during passive head motion enhancing both VOR gain and compensatory saccade strategies.

## Author contributions

NL, SK, CS, KJ, and CR did study conception. SK performed the rehabilitation. AK and CR collected the data. NL, AK, MH, and CR analyzed and interpreted the data. NL, ES, and CR contributed to the statistical analysis. MH and NL drafted the initial manuscript. NL, MH, and CR revised the manuscript. All authors have read and approved the final manuscript.

### Conflict of interest statement

ES is general manager and a shareholder of EyeSeeTec GmbH. NL is a shareholder and paid consultant to EyeSeeTec GmbH. CR was an employee of EyeSeeTec GmbH. The remaining authors declare that the research was conducted in the absence of any commercial or financial relationships that could be construed as a potential conflict of interest.

## Abbreviations

BVH: bilateral vestibular hypofunction
EMO: eye-movement-only rehabilitation
HITD-FT: head impulse testing device—functional test
HME: head-movement-emphasized rehabilitation
MDC: minimal detectable change
VOR: vestibulo-ocular reflex.
