# Supplementary material for: Head-Movement-Emphasized Rehabilitation in Bilateral Vestibulopathy
Source: Front Neurol. 2018 Jul 17;9:562. doi: 10.3389/fneur.2018.00562 (PMC6057116; doi:10.3389/fneur.2018.00562)
Supplement: Supplementary file 1 [file Data_Sheet_2.PDF]

Supplementary Material

Head-movement-emphasized rehabilitation in bilateral vestibulopathy

Nadine Lehnen[1,2,3], Silvy Kellerer[2], Alexander G. Knorr[4,5], Cornelia Schlick[2], Klaus Jahn[2,6], Erich Schneider[3], Maria Heuberger[2,7,*,#], Cecilia Ramaioli[2,3,#]

[1] Department of Psychosomatic Medicine and Psychotherapy, Klinikum rechts der Isar, Technical University of Munich, Munich, Germany

[2] German Center for Vertigo and Balance Disorders, Ludwig Maximilians University, Munich, Germany

[3] Institute of Medical Technology, Brandenburg University of Technology, Cottbus-Senftenberg, Germany

[4] Center for Sensorimotor Research, Ludwig Maximilians University, Munich, Germany

[5] Institute for Cognitive Systems, Department of Electrical and Computer Engineering, Technical University of Munich, Munich, Germany

[6] Department of Neurology, Schoen Clinic Bad Aibling, Bad Aibling, Germany

[7] Department of Neurology, Ludwig Maximilians University, Munich, Germany

*** Correspondence:**
Maria Heuberger
Maria.Heuberger@med.uni-muenchen.de

#These authors contributed equally to the work

Supplement 1: Rehabilitation programs

During both rehabilitation programs, patients practiced at home for about eight minutes five times a day for four weeks. The home exercise programs for both groups combined exercise description and diary and the patients ticked off the items after exercising.

1 Eye-movement-only rehabilitation (EMO, control intervention)

For the EMO program, an experienced physiotherapist told patients to keep the head absolutely still and to just move the eyes during exercises and to move their head as little as possible during daily life.

Week 1:

- Smooth pursuit: following the own thumb moved at arm-length horizontally and vertically with the eyes (sitting position, 1-2 min).
- Alternating fixating a near target (thumb held at arm-length) and a target 4 m away (metronome, 150-180 bpm, sitting position, 1-2 min).

- Eye movements to different targets within the room (metronome, 150-180 bpm, sitting position, 1-2 min).
- Five targets on a wall. Close eyes – fixate on target – close eyes – another target.
- Eye movements between two bottles on a table (sitting position, 1-2 min).

Week 2:

- Smooth pursuit: following a play card moved at arm-length horizontally and vertically with the eyes (sitting position, 1-2 min).
- Alternating fixating a near target (play card held at arm-length) and a target 4 m away (metronome, 150-180 bpm, sitting position, 1-2 min).
- Eye movements to different targets within the room (metronome, 150-180 bpm, sitting position, 1-2 min).
- Five targets on a wall. Close eyes – fixate on target – close eyes – another target.
- Eye movements between two bottles on a table (sitting position, 1-2 min).

Week 3:

- Smooth pursuit: following a picture with a face moved at arm-length horizontally and vertically with the eyes (sitting position, 1-2 min).
- Alternating fixating a near target (picture with a face held at arm-length) and a target 4 m away (metronome, 150-180 bpm, sitting position, 1-2 min).
- Eye movements to different targets within the room (metronome, 150-180 bpm, sitting position, 1-2 min).
- Five targets on a wall. Close eyes – fixate on target – close eyes – another target.
- Eye movements between two bottles on a table (sitting position, 1-2 min).

Week 4:

- Smooth pursuit: following a picture with a face moved at arm-length horizontally and vertically with the eyes (sitting position, 1-2 min)
- Alternating fixating a near target (picture with a face) and a target 4 m away (metronome, 150-180 bpm, sitting position, 1-2 min).
- Eye movements to different targets within the room (metronome, 150-180 bpm, sitting position, 1-2min).
- Five targets on a wall. Close eyes – fixate on target – close eyes – another target.
- Eye movements between two bottles on a table (sitting position, 1-2 min).

2 B) Head-movement-emphasized rehabilitation (HME, experimental intervention)

For HME, patients were instructed to move their head as often as possible during exercises and to perform large gaze shifts during daily life.

Week 1:

- Horizontal, vertical and torsional head movements to metronome sound (150-180 bpm) while fixating the own thumb placed in center held at arm-length (sitting position, 1-2 min).
- Horizontal, vertical and torsional head movements to metronome sound (150-180 bpm) while fixating a point placed in center 4 m away (sitting position, 1-2 min).

- Large natural combined eye-head gaze shifts between two targets from one top corner of the room to another next to it (sitting position, 1-2 min).
- Large natural combined eye-head gaze shifts between two targets from floor to the top of a door (standing in the door, 1-2 min).
- Large natural combined eye-head gaze shift from between two bottles on a table (sitting position, 1-2 min).

Week 2:

- Horizontal, vertical and torsional head movements to metronome sound (150-180 bpm) while fixating a play card placed in center held at arm-length (sitting position, 1-2 min).
- Horizontal, vertical and torsional head movements to metronome sound (150-180 bpm) while fixating a play card placed in center 4 m away (sitting position, 1-2 min).
- Large natural combined eye-head gaze shifts between two targets from one top corner of the room to the bottom one next to it, then the diagonally opposite (sitting position, 1-2 min).
- Large natural combined eye-head gaze shifts between two targets from floor to the top of a door (standing in the door, 1-2 min).
- Large natural combined eye-head gaze shift from between two bottles on a table (sitting position, 1-2 min).

Week 3:

- Horizontal, vertical and torsional head movements to metronome sound (150-180 bpm) while fixating a picture with a face placed in center held at arm-length (sitting position, 1-2 min).
- Horizontal, vertical and torsional head movements to metronome sound (150-180 bpm) while fixating a picture with a face placed in center 4 m away (sitting position, 1-2 min).
- Large natural combined eye-head gaze shifts between two targets from one top corner of the room to bottom one next to it, then the diagonally opposite (sitting position, 1-2 min).
- Large natural combined eye-head gaze shifts between two targets from floor to the top of a door (standing in the door, 1-2 min).
- Large natural combined eye-head gaze shift from between two bottles on a table (sitting position, 1-2 min).

Week 4:

- Horizontal, vertical and torsional head movements to metronome sound (150-180 bpm) while fixating a point on a moving background (e.g., on a window, placed in center, distance: arm-length, sitting position, 1-2 min).
- Horizontal, vertical and torsional head movements to metronome sound (150-180 bpm) while fixating a point on a moving background placed in center 4 m away (e.g., on a window, sitting position, 1-2 min).
- Large natural combined eye-head gaze shifts between two targets from one top corner of the room to the bottom one next to it, then the diagonally opposite (sitting position, 1-2 min).
- Large natural combined eye-head gaze shifts between two targets from floor to the top of a door (standing in the door, 1-2 min).
- Large natural combined eye-head gaze shift from between two bottles on a table (sitting position, 1-2 min).
